# 3D-printed ultra-small Brownian viscometers

**DOI:** 10.1038/s41598-024-64792-0

**Published:** 2024-06-17

**Authors:** Gaszton Vizsnyiczai, Jana Kubacková, Gergely T. Iványi, Cyril Slabý, Denis Horváth, Andrej Hovan, Alena Strejčková, Zoltán Tomori, Lóránd Kelemen, Gregor Bánó

**Affiliations:** 1 HUN-REN Biological Research Centre, Szeged, Institute of Biophysics, Temesvári krt. 62, Szeged, 6726 Hungary; 2 Department of Biotechnology, University of Szeged, Közép Fasor 52, Szeged, 6726 Hungary; 3 Department of Biophysics, Institute of Experimental Physics SAS, Watsonova 47, 040 01 Košice, Slovak Republic; 4 Doctoral School of Multidisciplinary Medical Sciences, University of Szeged, Szeged, 6720 Hungary; 5 Department of Biophysics, Faculty of Science, P. J. Šafárik University in Košice, Jesenná 5, 041 54 Košice, Slovak Republic; 6 Center for Interdisciplinary Biosciences, Technology and Innovation Park, P. J. Šafárik University in Košice, Jesenná 5, 041 54 Košice, Slovak Republic; 7 Department of Chemistry, Biochemistry and Biophysics, University of Veterinary Medicine and Pharmacy in Košice, Komenského 73, 041 81 Košice, Slovak Republic

**Keywords:** Viscometer, Brownian fluctuations, Power spectral density, Two-photon polymerization, Viscoelastic polymer nanowire, Lithography, Sensors and biosensors, Rheology

## Abstract

Measuring viscosity in volumes smaller than a microliter is a challenging endeavor. A new type of microscopic viscometers is presented to assess the viscosity of Newtonian liquids. Micron-sized flexible polymer cantilevers are created by two-photon polymerization direct laser writing. Because of the low stiffness and high elasticity of the polymer material the microcantilevers exhibit pronounced Brownian motion when submerged in a liquid medium. By imaging the cantilever’s spherically shaped end, these fluctuations can be tracked with high accuracy. The hydrodynamic resistance of the microviscometer is determined by fitting the power spectral density of the measured fluctuations with a theoretical frequency dependence. Validation measurements in water-glycerol mixtures with known viscosities reveal excellent linearity of the hydrodynamic resistance to viscosity, allowing for a simple linear calibration. The stand-alone viscometer structures have a characteristic size of a few tens of microns and only require a very basic external instrumentation in the form of microscopic imaging at moderate framerates (~ 100 fps). Thus, our results point to a practical and simple to use ultra-low volume viscometer that can be integrated into lab-on-a-chip devices.

## Introduction

The development of new concepts for downscaling viscosity measurements, i.e., the construction of ultra-low volume viscometers, has significance for a variety of industrial branches and applications. In pharmacology, for example, injectable drug formulations can be mentioned. Notably, decreasing the sample volumes required for viscosity measurements of therapeutic (e.g., monoclonal antibody) formulations significantly reduces the experimental demands on expensive samples^1–3^. Several micro-rheological techniques have been developed in the past to address the problem of small-volume viscosity measurements. Review papers and books provide a summary of the available experimental approaches^4–9^. Methods for probing viscosity using individual micron-sized solid objects, which are thus relevant to the current work, are reviewed below.

The first group of micro-rheological techniques is based on the motion of micron-sized beads dispersed in the liquid medium. The Brownian motion of freely moving spherical particles is followed by video-tracking in the simplest case. The particle diffusion coefficient is determined, and the corresponding viscosity is calculated using the Stokes–Einstein relation^10–12^. Another set of experimental methods makes use of optical tweezers^13,14^. Optical traps keep the beads in a static harmonic potential. In this passive regime, the medium viscosity is assessed from the Brownian fluctuations of the beads around the equilibrium position by measuring either the power spectrum^15–17^ or the position autocorrelation function^17,18^ of the stochastic fluctuations. Alternatively, optical forces can be used to actively induce bead motion. In this case, the viscosity is determined by measuring the response of trapped particles to oscillatory drag forces. The given experimental designs work with single-trap^19–21^, or dual-trap^22,23^ configurations. Viscosity measurements based on optically trapped spherical beads rely, in most cases, on the bead displacement calibration and the knowledge of the trap stiffness. The trap stiffness can be calibrated using the energy equipartition at thermal equilibrium^24^ (the local temperature must be known here).

The second large group of micro-rheological techniques employs vibrating cantilevers immersed in liquid. AFM (atomic force microscopy) cantilevers of ca. 100–400 µm length are commonly used. The viscosity and the density of the medium are determined by evaluating the resonant frequency (typically in the range of 10^3^–10^5^ Hz) and the quality factor of the oscillators^25^. Cantilever vibrations are stimulated either actively, via external actuation^26–30^, or passively, via thermal fluctuations^25,31,32^. Different MEMS-based cantilever-type viscosity sensors were also reported^33^.

**Figure 1:**
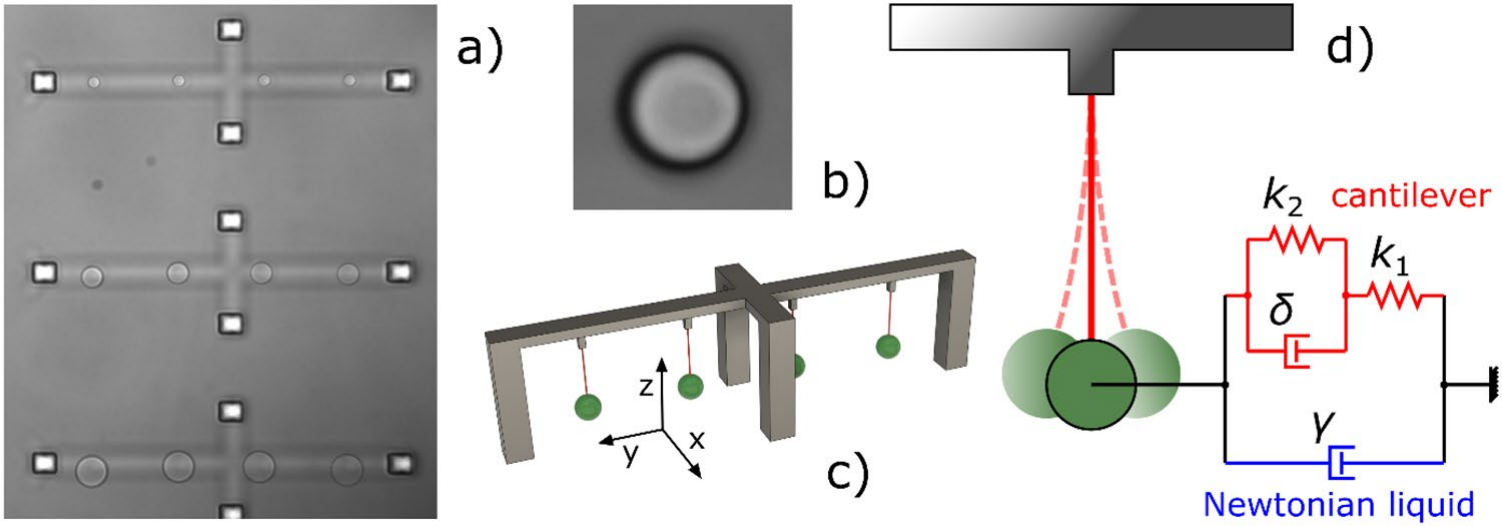
The viscometer structure. (**a**) Brightfield image of microstructures with beads of 2 µm, 4 µm, and 6 µm radius. (**b**) A screenshot from the video (see the Supplementary Materials) shows the 4 µm radius bead. (**c**) Schematic view of the solid support with four vertical cantilevers. A spherical bead is attached to each nanowire beam. (**d**) The mechanical model of the viscometer with the viscoelastic beam and the microbead immersed in a Newtonian liquid.

In this work, we introduce a new type of microscopic viscometer whose working principle is based on the passive Brownian motion of a low-stiffness flexible microcantilever. The microcantilever is created by two-photon polymerization microfabrication, with a support microstructure anchored to a microscope coverglass. A micron-sized spherical bead is formed on the cantilever's free end, allowing for highly accurate imaging-based tracking of its thermal fluctuations in a liquid medium (Fig. 1). The viscosity of the surrounding fluid is then obtained using the power spectral density of the measured cantilever fluctuations. The micro-viscometers were tested and validated in water-glycerol mixtures of known viscosity.

## Materials and methods

### Viscometer fabrication

OrmoComp photoresist and OrmoDev developer were purchased from Micro Resist Technology. SylgardTM 184 from Dow Corning GmbH, Germany was used to prepare PDMS layers. High-purity distilled water was used to prepare water-glycerol mixtures of known viscosity. The viscosity of the mixtures was calculated using an empirical formula^34^.

The flexible structures were fabricated by TPP-DLW (two-photon polymerization direct laser writing) of OrmoComp using a 785 nm, 100 MHz femtosecond laser (C-Fiber 780, Menlo Systems, Germany). A 40 × oil-immersion objective (NA 1.2, C PlanApo, Zeiss, Germany) was applied to focus the laser beam into a photoresist droplet, which was placed on top of a 0.17 mm thick glass coverslip. A closed channel with two inlets made of 4 mm thick PDMS block was formed over the droplet. An acousto-optic modulator (APE 130,101, APE GmbH, Germany) adjusted the laser intensity. The laser focus was scanned by moving the sample with a piezo stage (P-563.3CL PIMars, Physik Instrumente, Germany) in three directions. The microstructure design is shown in Fig. 1. Four identical cantilevers were placed on a single support. The nanowire beams were drawn vertically as a single line, and their stiffness was adjusted by setting the scanning speed and the laser power. Optimal conditions, ensuring both flexibility and self-support, were obtained at 10 μm/s scan speed and 5 mW laser power. After the polymerization process, the uncured photoresist was washed away by filling the channel few times with OrmoDev developer and ethanol. Just before developer-ethanol exchange, the coverslip was irradiated with the mercury lamp of a fluorescence microscope (HBO 50) to finish the polymerization and stabilize the microstructures^35^. Finally, the microfluidic channel was filled with distilled water.

Microstructures with designed bead radius, *R*_d_, of 2 µm, 4 µm, and 6 µm were tested. The length of the cantilever was 20 µm in all cases. The sphere centre was positioned at 15 µm above the coverslip surface. The typical diameter of the used cantilevers was 0.35 $\mu$m.

### Material properties of photopolymer microstructures

The elastic properties of TPP-DLW-fabricated microstructures have already been investigated. Displacement recovery measurements of stretched coil-shaped micro-springs made from SCR500 and PMMA photoresins were reported^35–37^. Most importantly, the bulk and microscopic shear moduli of the photopolymer material were found to differ significantly, with microscopic values being orders of magnitude lower in some cases. The Young’s modulus of photo-polymers in micron-sized objects were measured by deflection tests using nano-indentation equipment^38^. The elastic moduli mentioned thus far only describe the (near-static) slow motion of photopolymer microstructures. When operating the microstructures in a dynamic regime involving Brownian fluctuations, the viscoelasticity of the photopolymer must be considered.

The micro-viscometers investigated in this study are made of OrmoComp, a bio-compatible photoresist material with exceptional flexibility^39^. Our previous work investigated the viscoelastic properties of OrmoComp nanowire cantilevers by measuring the recovery motion of structures deflected by an optical tweezer setup^40,41^. It was shown that the cantilever mechanics can be described by a three-parameter viscoelastic model. Furthermore, an analytical solution for the power spectral density of freely fluctuating viscoelastic cantilever/microsphere structures immersed in Newtonian liquids was obtained^42^. After being refined, this result serves as a theoretical basis for the presented viscometers.

### Fluctuation measurements

The viscometers submerged to different water/glycerol mixtures were viewed in brightfield under a microscope equipped with a camera operated at 663 fps. Brownian fluctuations were captured by taking 30-s videos. The viscometer fluctuations were assessed by image tracking the bead position. We used a custom MATLAB algorithm that employed a direct search scheme to determine the spatial shift of the bead on each recorded image with respect to the first recorded image. Using normalized cross correlation (normxcorr2 MATLAB function), the algorithm first found the spatial shift with 1 pixel resolution. In the next step the algorithm probed half pixel shifts in each direction added over the already known 1 pixel resolution shifts. For this, we spatially shifted the image with fast Fourier transform, using Fourier Shift Theorem, and calculated the mean absolute pixelwise difference between the shifted images and the reference image. The algorithm then selected the smallest mean difference value among the nine half pixel shift combinations (for directions: ± X and ± Y, and 0 shift) and repeated the search probing quarter pixel shifts. This step was repeated eight times to achieve a resolution of ${2}^{-8}\approx 0.004$ pixels, corresponding to 0.34 nm. Only the one-dimensional data along the Y axis (see Fig. 1) were used for further analysis.

### Data analysis

We begin to derive the theoretical background of the current viscometer operation by defining the fundamental postulates that mark the model framework. The first assumption concerns the viscometer inertia. The effective Young’s modulus of polymer nanowires is four orders of magnitude lower than that of silicon, which is commonly used in AFM and/or MEMS cantilever applications. The stiffness of nanowires employed in our viscometers is extremely low, on the order of 0.001 pN/nm^41^. At the same time, because of significant dissipation caused by the surrounding liquid and the cantilever material's internal viscoelasticity, low frequency motion dominates the system dynamics. The cantilever and the attached bead motion is strongly overdamped with no resonances observed in the studied low-frequency range. Due to these facts, all inertial forces and the related effects are neglected in our study. Owing to the cylindrical cantilever shape, the two possible oscillation directions X and Y are assumed to be independent and identical. The second assumption concerns the viscometer geometry. The external viscous damping force of the surrounding liquid is assumed to act primarily on the attached bead, representing the thin nanowire limit. This latter assumption will be refined later by using an empirical formula to correct the model for the finite cantilever thickness.

Figure 1d depicts the mechanical scheme of the micro-bead attached to the viscoelastic cantilever; only a single oscillation direction is considered. The nanowire viscoelasticity is characterized by the upper (red) arm, which evolves from the Kelvin form of a three-parameter standard linear solid^40,41^ with two spring constants (stiffnesses) *k*_1_, *k*_2_, and a single internal damping coefficient *δ*. The lower (blue) arm containing the hydrodynamic resistance *γ* represents the Newtonian liquid that damps the micro-bead fluctuations. Based on this model, and taking the previous assumptions into account, the power spectrum of the observed Brownian fluctuations obeys a double Lorentzian distribution^42^:

1 $${P}_{(f)}=\frac{{k}_{\text{B}}T}{2{\pi }^{2}\gamma \left({f}_{\text{c}2}^{2}-{f}_{\text{c}1}^{2}\right)}\left(\frac{{\frac{{K}_{\text{A}}}{4{\pi }^{2}} - f}_{\text{c}1}^{2}}{{f}_{\text{c}1}^{2}+{f}^{2}}\text{+} \, \frac{{f}_{\text{c}2}^{2} - \frac{{K}_{\text{A}}}{4{\pi }^{2}}}{{f}_{\text{c}2}^{2}+{f}^{2}}\right)$$

The two corner frequencies *f*_c1_, *f*_c2_, as well as the auxiliary *K*_A_ parameter are non-trivial functions of the three viscoelastic parameters (*k*_1_, *k*_2_, *δ*) and the hydrodynamic resistance γ (see the Supplementary Materials; for more details we refer to^42^). In the frequency region above the two corner frequencies, the power spectrum simplifies significantly:

2 $${P}_{(f\gg {f}_{\text{c}1},{f}_{\text{c}2})}=\frac{{k}_{\text{B}}T}{2{\pi }^{2}\gamma } \frac{1}{{f}^{2}}$$

The criterion to reach pure 1/*f*^*2*^ dependence is to stay at least one order of magnitude above the two corner frequencies. This frequency region is identical to the high-frequency power spectrum limit of a bead trapped in a harmonic potential^15^ and reflects free diffusive Brownian motion^43^. We emphasize that the expression of Eq. 2 is independent of the cantilever viscoelastic properties; the hydrodynamic resistance *γ* is the only structure-related parameter that affects the fluctuations. As *γ* is proportional to the solution viscosity *η*, the amplitude of the power spectral density in the 1/*f*^2^ regime can be used for viscosity measurements. A single calibration parameter, *ξ*_cal_, is introduced to relate the solution viscosity to the measured hydrodynamic resistance:

3 $$\eta ={\xi }_{\text{cal }}\gamma$$

In practice, a calibration measurement in a liquid with known viscosity (*η*_ref_) can determine *ξ*_cal_ for a specific viscometer structure: *ξ*_cal_ = *η*_ref_/*γ*_ref_.

Two additional effects are considered when fitting the experimental power spectral density data in the theoretical 1/*f*^2^ region. First, due to finite sampling frequency the experimental spectra are aliased near the Nyquist frequency^15^. Second, video capture introduces a blur effect that needs to be corrected^44,45^. Fitting the experimental power spectral density data by the corrected theoretical function is required for further data analysis. To reduce experimental noise in the power spectral density, the Welch method with *n* = 32 non-overlapping time windows is used. The experimental curves are analyzed using least-square fitting in the 20–90 Hz spectral range, with theoretical values used as weights (for details we refer to Ref. 46). Finally, the obtained power spectra are corrected for the bias introduced by least-square fitting when combined with spectral averaging^46^.

## Results

**Figure 2:**
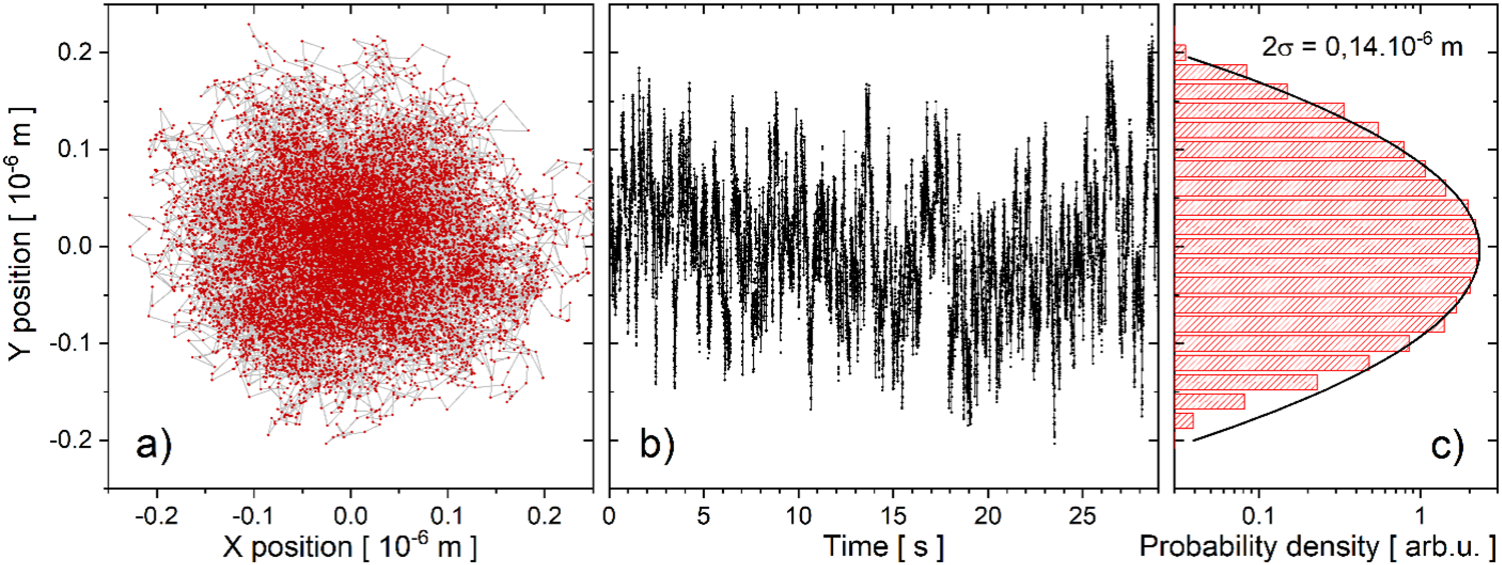
Brownian fluctuations of the microbead attached to polymer nanowire. (**a**) Typical record of the *R*_d_ = 4 µm bead center position in water as tracked in the X–Y plane. (**b**) The time course of the bead center fluctuations along the Y coordinate during the 30 s data acquisition period. (**c**) The probability density of the bead center position fitted by a Gaussian distribution (σ is the standard deviation).

Figure 2a depicts typical fluctuations of microcantilever structures immersed in water. The observed pattern has a minor asymmetry, as shown in Figure S1 of the Supplementary Materials, which depicts the position probability distributions for the X and Y directions. The difference in distribution widths (around 13%) is probably due to the inherent asymmetry of the polymerization laser beam, which is then transferred to the cantilever. The time course of the bead fluctuations along the Y axis (plotted in Fig. 2b) is used for data analysis. The probability density of the bead center position is fitted by a Gaussian distribution (Fig. 2c) as expected for Boltzmann statistics. The deviation from the Gaussian distribution at the tails (see also Figure S1) is most likely caused by a minor drift in the camera image. However, a low frequency drift has no effect on the fluctuation power spectral densities in the frequency range used for data analysis (see below). Using the equipartition theorem, the distribution variance is used to calculate an effective trapping potential stiffness^44^. The obtained value, *k*_eff_ = 0.8 10^−6^ Nm^−1^ is of the same order as the equilibrium stiffness reported previously for similar viscoelastic cantilevers^41^. The Supplementary Materials include a video of a fluctuating 4 µm radius bead.

**Figure 3:**
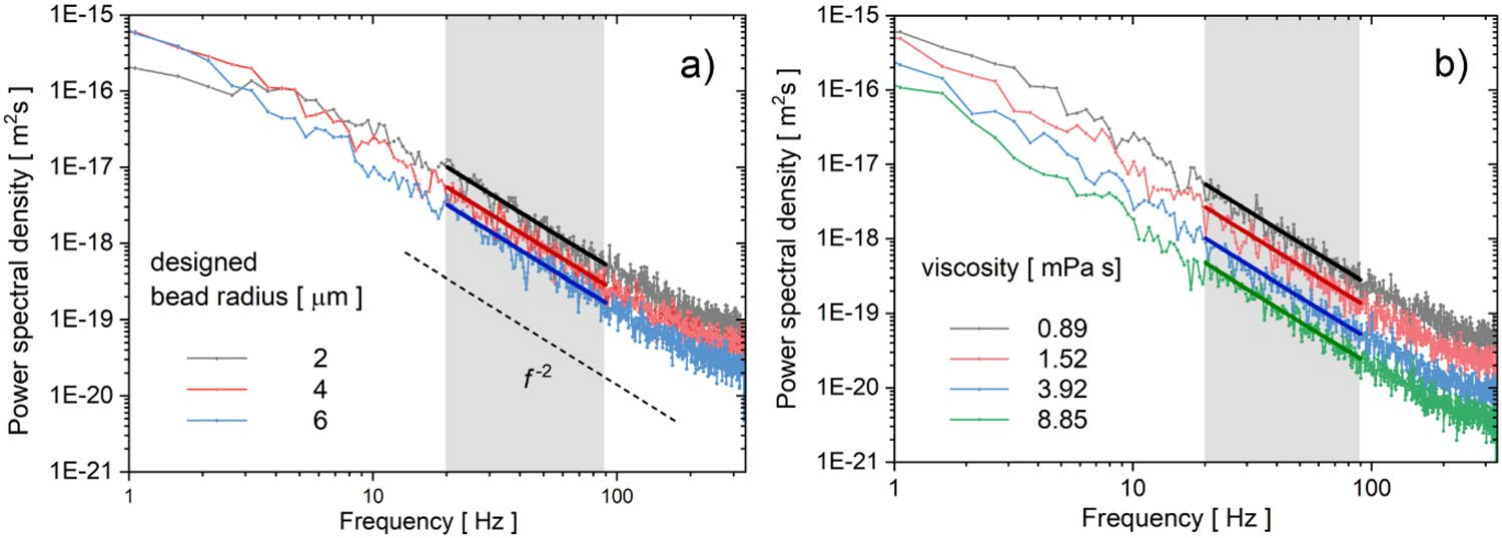
Power spectral densities (PSD) of the bead position fluctuations. (**a**) Typical PSDs measured in pure water using microstructures with designed bead radii of 2 µm, 4 µm, and 6 µm. The shaded area indicates the 20–90 Hz region used for fitting the spectra. The fitted curves are plotted as solid lines. The dashed curve shows a 1/*f*^2^ dependence. (**b**) PSDs obtained in different water-glycerol mixtures using an *R*_d_ = 4 µm bead. The legend indicates the solution viscosities.

Figure 3a depicts the measured power spectral densities for three different bead radii (*R*_d_ = 2 µm, 4 µm, and 6 µm). The power spectra are relatively noisy in the low-frequency region, making the data fitting with double-Lorentzian functions over the entire frequency range difficult (see Eq. 1). However, determining the parameters of the double-Lorentzian fits is not the goal of this work. Rather, we focus on the frequency region (above 20 Hz) where theoretical power spectra decay with the second power of the frequency (see Eq. 2 and the dashed line in Fig. 3a). The higher the bead radius, the lower the power spectral density in this region. Due to aliasing, the measured power spectra level off near the Nyquist frequency (332 Hz).

Figure 3b depicts power spectra measured with a *R*_d_ = 4 µm bead in various water-glycerol mixtures. A higher viscosity (higher glycerol content) decreases the power spectral density in the 1/*f*^2^ domain. This effect can be used to measure viscosity, as shown below.

**Figure 4:**
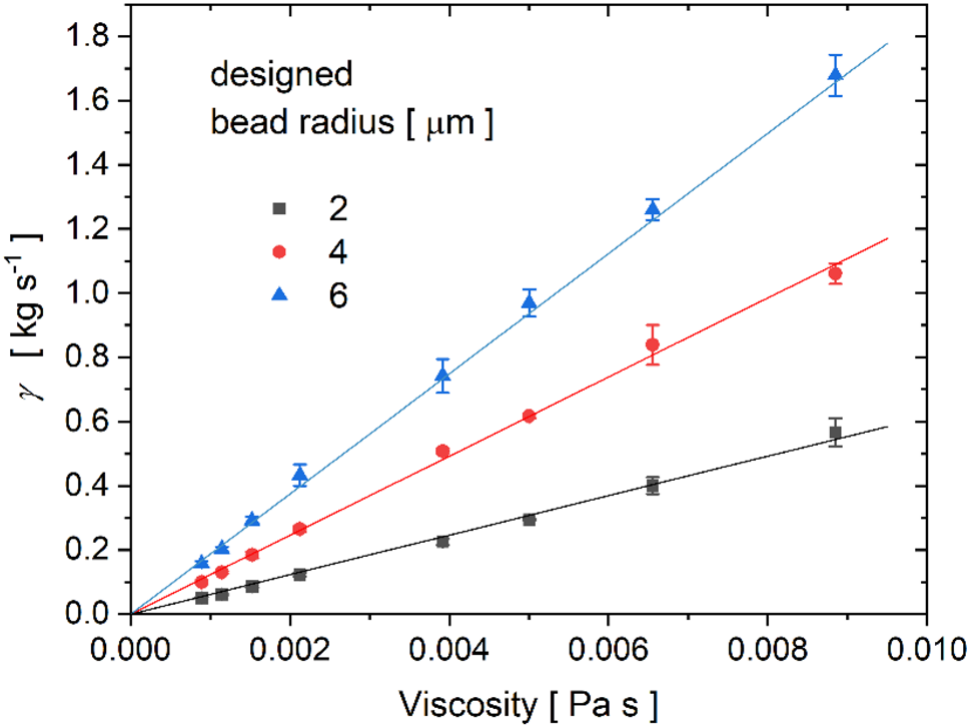
Viscosity measurements. The hydrodynamic resistance values obtained with microstructures immersed in different water-glycerol mixtures plotted against the solution viscosity. All the measurements were repeated with three different microstructures, and the error bars represent the errors of their mean *γ* values.

The power spectral data were fitted in the frequency range 20–90 Hz, as indicated by the grey regions in Fig. 3. The hydrodynamic resistance *γ* obtained from the fit for different solution viscosities and different bead radii is plotted in Fig. 4. As expected, the measured *γ* values exhibit a linear dependence on the solution viscosity and can therefore be accurately fitted by linear curves that pass through the origin. The experimental points are well aligned along the fitted curves, which paves the way for viscosity measurement applications and allows for a simple calibration procedure. The slope of the fitted curves equals the calibration constant reciprocal, 1/*ξ*_cal_ (see Eq. 3). In principle, a single measurement made in a solution of known viscosity is sufficient to obtain the value of *ξ*_cal_ for a specific microstructure type. Larger bead radii yield smaller calibration constants. In the Supplementary Materials we provide a more detailed analysis of the relationship between the calibration constant and the bead radius.

## Discussion

Flexible stand-alone microstructures composed of a nanowire cantilever with a spherically shaped free end were introduced to measure viscosity of sub-microliter volume samples. The power spectral density of the microbead Brownian fluctuations decays with the second power of the frequency in a region spanning more than one order of magnitude, which is consistent with the proposed theoretical model. As expected, good linearity was found between the hydrodynamic resistance obtained by fitting the power spectra and the solution viscosity. The microstructures were calibrated using the known viscosity of water-glycerol mixtures.

The primary benefit of evaluating power spectral densities in the 1/*f*^2^ domain is that the data in this region are unaffected by microstructure mechanical properties (see Eq. 2). This is especially significant when we consider that the mechanical properties of photopolymer microstructures can change in different environments due to the penetration of solute molecules into the polymer pores, as previously demonstrated for aqueous glucose solutions^41^. Fortunately, the 1/*f*^2^ region can be easily reached using the low-stiffness flexible cantilevers presented in this work. Surface adsorption of analytes should not interfere with measurements if the structure dimensions (bead diameter) do not change dramatically. It was found sufficient to evaluate data in the 20–90 Hz range, where the limiting power spectral density defined by Eq. 2 is well above the noise level even at the highest viscosity studied. The 30-s videos recorded during our measurements represent a good balance between the required data storage capacity and measurement time and the good quality fits to the power spectral density data.

The Brownian micro-viscometers presented in our work have a different operation regime than other viscometer types that rely on thermal fluctuations. In comparison to microbeads trapped in optical tweezers, a simpler optical setup consisting of a microscope equipped with a fast camera is all that is required to measure viscosity. Furthermore, the current microstructures are prepared at well-defined positions on the substrate, e.g., in microfluidic channels, so there is no need to disperse them into small-volume samples; the structures are easy to localize under the microscope. In comparison to AFM and MEMS cantilevers, we highlight the small dimensions of the polymer structures. The stand-alone viscometers tested here have a characteristic size of ~ 30 µm, which can be further reduced. There are no additional (bulky) supporting parts required to operate the viscometers. In principle, sample volumes well below one microliter are possible and sufficient for viscosity measurements. Some technical issues must be resolved to turn the current structures into a commercial product. One possible development path points to disposable chips with pre-fabricated microcantilever viscometer structures.

### Supplementary Information

Supplementary Video 1.

Supplementary Information 1.

## Data Availability

The datasets generated during and/or analyzed during the current study are available from the corresponding authors on reasonable request.

## References

[1] Jezek J (2011). Viscosity of concentrated therapeutic protein compositions. Adv. Drug Deliv. Rev..

[2] Shire SJ, Shahrokh Z, Liu J (2004). Challenges in the development of high protein concentration formulations. J. Pharm. Sci..

[3] Zhang ZH, Liu Y (2017). Recent progresses of understanding the viscosity of concentrated protein solutions. Curr. Opin. Chem. Eng..

[4] Gupta S, Wang WS, Vanapalli SA (2016). Microfluidic viscometers for shear rheology of complex fluids and biofluids. Biomicrofluidics.

[5] Xia Q, Xiao H, Pan Y, Wang L (2018). Microrheology, advances in methods and insights. Adv. Colloid Interface Sci..

[6] Waigh TA (2016). Advances in the microrheology of complex fluids. Rep. Progress Phys..

[7] Furst EM, Squires TM (2017). Microrheology. Microrheology.

[8] Liu W, Wu C (2018). Rheological study of soft matters: A review of microrheology and microrheometers. Macromol. Chem. Phys..

[9] Cicuta P, Donald AM (2007). Microrheology: A review of the method and applications. Soft Matter..

[10] Sie Y-S, Chuang H-S (2014). A micro-volume viscosity measurement technique based on mu PIV diffusometry. Microfluid. Nanofluid..

[11] Clayton KN, Lee D, Wereley ST, Kinzer-Ursem TL (2017). Measuring biotherapeutic viscosity and degradation on-chip with particle diffusometry. Lab Chip.

[12] Josephson LL, Furst EM, Galush WJ (2016). Particle tracking microrheology of protein solutions. J. Rheol..

[13] Brau RR (2007). Passive and active microrheology with optical tweezers. J. Opt. A-Pure Appl. Opt..

[14] Yao A, Tassieri M, Padgett M, Cooper J (2009). Microrheology with optical tweezers. Lab Chip.

[15] Berg-Sorensen K, Flyvbjerg H (2004). Power spectrum analysis for optical tweezers. Rev. Sci. Instrum..

[16] Pesce G (2009). Microrheology of complex fluids using optical tweezers: A comparison with macrorheological measurements. J. Opt. A-Pure Appl. Opt..

[17] Keen S (2009). Multipoint viscosity measurements in microfluidic channels using optical tweezers. Lab Chip.

[18] Tassieri M (2015). Microrheology with optical tweezers: Measuring the relative viscosity of solutions ‘at a glance’. Sci. Rep..

[19] Neckernuss T (2016). Active microrheology with optical tweezers: a versatile tool to investigate anisotropies in intermediate filament networks. J. Phys. D-Appl. Phys..

[20] Statsenko A, Inami W, Kawata Y (2017). Measurement of viscosity of liquids using optical tweezers. Opt. Commun..

[21] Robertson-Anderson RM (2018). Optical tweezers microrheology: from the basics to advanced techniques and applications. Acs Macro Lett..

[22] Lamperska W, Masajada J, Drobczynski S, Gusin P (2017). Two-laser optical tweezers with a blinking beam. Opt. Lasers Eng..

[23] Paul S, Kumar R, Banerjee A (2018). Two-point active microrheology in a viscous medium exploiting a motional resonance excited in dual-trap optical tweezers. Phys. Rev. E.

[24] Tassieri M (2019). Microrheology with optical tweezers: Peaks & troughs. Curr. Opin. Colloid Interface Sci..

[25] Boskovic S, Chon JWM, Mulvaney P, Sader JE (2002). Rheological measurements using microcantilevers. J. Rheol..

[26] Schilowitz AM, Yablon DG, Lansey E, Zypman FR (2008). Measuring hydrocarbon viscosity with oscillating microcantilevers. Measurement.

[27] Bircher BA, Krenger R, Braun T (2016). Automated high-throughput viscosity and density sensor using nanomechanical resonators. Sens. Actuators B-Chem..

[28] Cakmak O, Ermek E, Kilinc N, Yaralioglu GG, Urey H (2015). Precision density and viscosity measurement using two cantilevers with different widths. Sens. Actuators A-Phys..

[29] Bergaud C, Nicu L (2000). Viscosity measurements based on experimental investigations of composite cantilever beam eigenfrequencies in viscous media. Rev. Sci. Instrum..

[30] Rust P, Cereghetti D, Dual J (2013). A micro-liter viscosity and density sensor for the rheological characterization of DNA solutions in the kilo-hertz range. Lab Chip.

[31] Hennemeyer M, Burghardt S, Stark RW (2008). Cantilever micro-rheometer for the dcharacterization of sugar solutions. Sensors.

[32] Paxman R, Stinson J, Dejardin A, McKendry RA, Hoogenboom BW (2012). Using micromechanical resonators to measure rheological properties and alcohol content of model solutions and commercial beverages. Sensors.

[33] Singh P, Sharma K, Puchades I, Agarwal PB (2022). A comprehensive review on MEMS-based viscometers. Sens. Actuators A-Phys..

[34] Cheng NS (2008). Formula for the viscosity of a glycerol–water mixture. Indus. Eng. Chem. Res..

[35] Nakanishi S, Shoji S, Kawata S, Sun H (2007). Giant elasticity of photopolymer nanowires. Appl. Phys. Lett..

[36] Sun HB, Takada K, Kawata S (2001). Elastic force analysis of functional polymer submicron oscillators. Appl. Phys. Lett..

[37] Ushiba S (2015). Size dependent nanomechanics of coil spring shaped polymer nanowires. Sci. Rep..

[38] Cicha K (2012). Young’s modulus measurement of two-photon polymerized micro-cantilevers by using nanoindentation equipment. J. Appl. Phys..

[39] Schizas C, Karalekas D (2011). Mechanical characteristics of an Ormocomp (R) biocompatible hybrid photopolymer. J. Mech. Behav. Biomed. Mater..

[40] Kubackova J (2020). Bending dynamics of viscoelastic photopolymer nanowires. Appl. Phys. Lett..

[41] Kubackova J (2021). Assessing the viscoelasticity of photopolymer nanowires using a three-parameter solid model for bending recovery motion. Nanomaterials.

[42] Bano G (2021). Power spectral density analysis of nanowire-anchored fluctuating microbead reveals a double lorentzian distribution. Mathematics.

[43] Lukic B (2007). Motion of a colloidal particle in an optical trap. Phys. Rev. E.

[44] Wong WP, Halvorsen K (2006). The effect of integration time on fluctuation measurements: Calibrating an optical trap in the presence of motion blur. Opt. Express.

[45] van der Horst A, Forde NR (2010). Power spectral analysis for optical trap stiffness calibration from high-speed camera position detection with limited bandwidth. Opt. Express.

[46] Norrelykke SF, Flyvbjerg H (2010). Power spectrum analysis with least-squares fitting: Amplitude bias and its elimination, with application to optical tweezers and atomic force microscope cantilevers. Rev. Sci. Instrum..

