# Supplementary material for: 3D-printed ultra-small Brownian viscometers
Source: Sci Rep. 2024 Jun 17;14:13964. doi: 10.1038/s41598-024-64792-0 (PMC11183119; doi:10.1038/s41598-024-64792-0)
Supplement: Supplementary file 2 — Supplementary Information 1. [file 41598_2024_64792_MOESM2_ESM.docx]

**Supplementary Material**

**3D-printed ultra-small Brownian viscometers**

Gaszton Vizsnyiczai, Jana Kubacková, Gergely T. Iványi, Cyril Slabý, Denis Horváth,

Andrej Hovan, Alena Strejčková, Zoltán Tomori, Lóránd Kelemen, Gregor Bánó

**Expressions for the *f*_c1_, *f*_c2_, and *K*_A_ parameters:**

The *K*_A_ parameter and the two corner frequencies *f*_c1_ and *f*_c2_ that occur in the double Lorentzian power spectral density function (see Equation 1 of the main text) can be calculated from the viscoelastic parameters of the cantilever structure (*k*_1_, *k*_2_, *δ*) and the hydrodynamic resistance *γ* as follows:

$K_{\text{A}}=\frac{\left( k_{1}+k_{2} \right)^{2}}{\delta^{2}}+\frac{k_{1}^{2}}{\gamma\delta}$ ,

$4\pi^{2}f_{\text{C1,2}}=\frac{K_{\text{C}}}{2}-K_{\text{B}}\pm\frac{1}{2}\sqrt{K_{\text{C}}\left( K_{\text{C}}-4K_{\text{B}} \right)}$,

where we introduced two additional parameters, *K*_B_ and *K*_C_:

$K_{\text{B}}=\frac{k_{\text{1}}k_{\text{2}}}{\gamma\delta}$,

$K_{\text{C}}=\left[ \frac{k_{2}}{\delta}+k_{1}\left( \frac{1}{\gamma}+\frac{1}{\delta} \right) \right]^{2}$.

For more information and the derivation of the above expressions, please see [1].

**The fluctuation pattern asymmetry**

Figure S1 compares the probability density function of the bead center position in the X and Y directions. A minor asymmetry is observed, with the cantilever fluctuating to a lesser extent in the Y direction. The difference in distribution widths (around 13%) is probably due to the inherent asymmetry of the polymerization laser beam, which is then transferred to the cantilever.

**
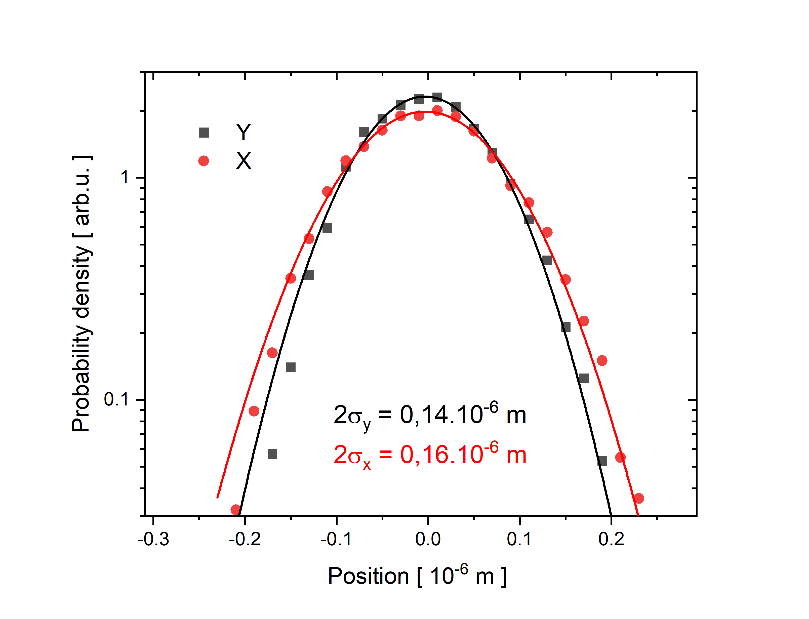
**

**Figure S1.** The probability density distributions in the X and Y directions as calculated for the fluctuation pattern plotted in Figure 2a. σ_x_ and σ_y_ are the standard deviations of the fitted Gaussian distributions.

**Estimating the effective hydrodynamic radius of the viscometer structures:**

In the paper main text, a single calibration parameter *ξ*_cal_ was introduced to relate the measured hydrodynamic resistance to the solution viscosity. Theoretical calculation of hydrodynamic resistance could also be used to avoid the need for calibration measurements and to determine the relationship between the viscometer dimensions (bead radius, cantilever length, and width) and the calibration parameter. However, this is difficult for the viscometer because it has a composite shape consisting of a cantilever connected to a microsphere located near a large surface (carrier coverglass) and a support structure. While the resistance of an isolated microsphere can be simply calculated using Stoke’s law, there is no theoretical formula for a rod-like cantilever with one end fixed. The presence of the substrate coverglass also introduces an increase in the hydrodynamic resistance. While this effect can be theoretically accounted for in the case of the microsphere using Faxen’s law [2], it is not possible to do so in the case of the cantilever. The proximity of the support structure and the neighboring beads may also increase the drag on the viscometer by an unknown factor. Due to these factors, the precise calculation of the hydrodynamic resistance of the viscometer is extremely complicated.

We can still gain a better understanding of the measurement data by using a simplified theoretical description that relies on a few assumptions. First, we assume that the interaction of the beads with the coverglass dominates the hydrodynamic coupling between the microstructure and the surrounding surfaces. We assume that the cantilever experiences a significantly lower drag increase because it is much smaller than the sphere and, on average, it is farther away from the glass. We also ignore the drag increase effect caused by the proximity of the support structure and other nearby viscometers, because they have significantly smaller surface areas than the carrier coverglass.

The hydrodynamic resistance of a microsphere in Newtonian liquid is given by the Stokes law: *γ*_sphere_=6π*ηR*, where *R* is the sphere radius. We introduce an effective radius *R*_eff_ that reflects the properties of the used microstructures while maintaining the simple expression for the hydrodynamic resistance: γ_(free viscometer)_=6π*ηR*_eff_. It is important to emphasize that the effective radius aims to describe the free-standing system of the bead connected to the cantilever, not affected by the presence of the nearby coverglass. It is advantageous to consider the relationship between the designed radius of the used microspheres *R*_d_ and the effective radius of the whole microstructure *R*_eff_ in the following form:

$R_{\text{eff}}={\alpha R}_{d}+C$.

The given empirical expression has the simplest linear form, which describes our experimental data presented below. The proportionality factor α expresses the distortions in shape and size of the used beads in relation to the designed spherical shape. The distortion (see the SEM images in Figure S3) is mostly caused by the elliptical voxel volume of the polymerization apparatus. The additive constant *C* represents the cantilever contribution.

The relation between the effective bead radius and the calibration constant *ξ*_cal_ is non-trivial and affected by the drag increase due to the nearby glass surface. As mentioned above, we only consider the hydrodynamic coupling between the bead and the coverglass, which can be analytically calculated using the Faxén correction formula [2]: FaxCorr=(1-(9/16)*(*αR*_d_/*d*)+1/8*(*αR*_d_/*d*)^3), where *d* is the distance between the sphere’s center and the coverglass surface. Taking all the assumptions into account we end up with the following relation:

$$\frac{1}{\xi_{cal}}=6\pi\left( \frac{\alpha R_{d}}{\mathrm{FaxCorr}}+C \right)$$

The nonlinearity of the Faxen formula makes it difficult to obtain a self-consistent analytical solution to the problem. However, we can iteratively recover *α* and *C* from the calibration constants measured for the three different designed radii used in the experiments. The iterative process includes the following steps. We start with the initial values of *α*=1 and *C*=0. In each iteration step we calculate the Faxén correction (for all three designed bead radii: *R*_d_=2,4 and 6 µm) using the actual *α* value. Then the following expression is evaluated to get the iterated effective radius *R*_eff_^it^:

$$R_{\mathrm{eff}}^{\mathrm{it}}=\left( \frac{1}{{6\pi\xi}_{cal}}-C \right)\mathrm{FaxCorr}+C$$

The three points obtained this way (and plotted against *R*_d_) are fitted with a linear dependence to get new iterated values for *α* and *C*. The described process converges within a few iterations; the results are shown in Figure S2.


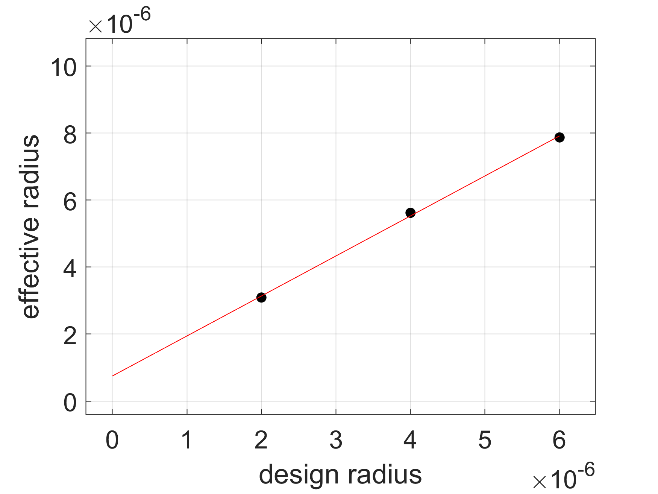


**Figure S2.** The effective radius plotted against the designed radius. The values are shown in [ m ] units.

The converged values of fitted parameters are: *α*=1.2 and *C* = 0.75 µm. These findings show that the bead radius exceeds its designed value, and the cantilever's contribution to the effective radius (represented by C) is not negligible.

**SEM imaging of the microviscometers**

SEM images of the microstructures are shown in Figure S3. The cantilevers were made thicker and shorter here, which reduced deformations caused by the drying process. Clearly, the microbeads have a non-spherical prolongated shape. The measured bead dimensions exceed the designed bead radius, which is consistent with the effective radius analysis presented above.


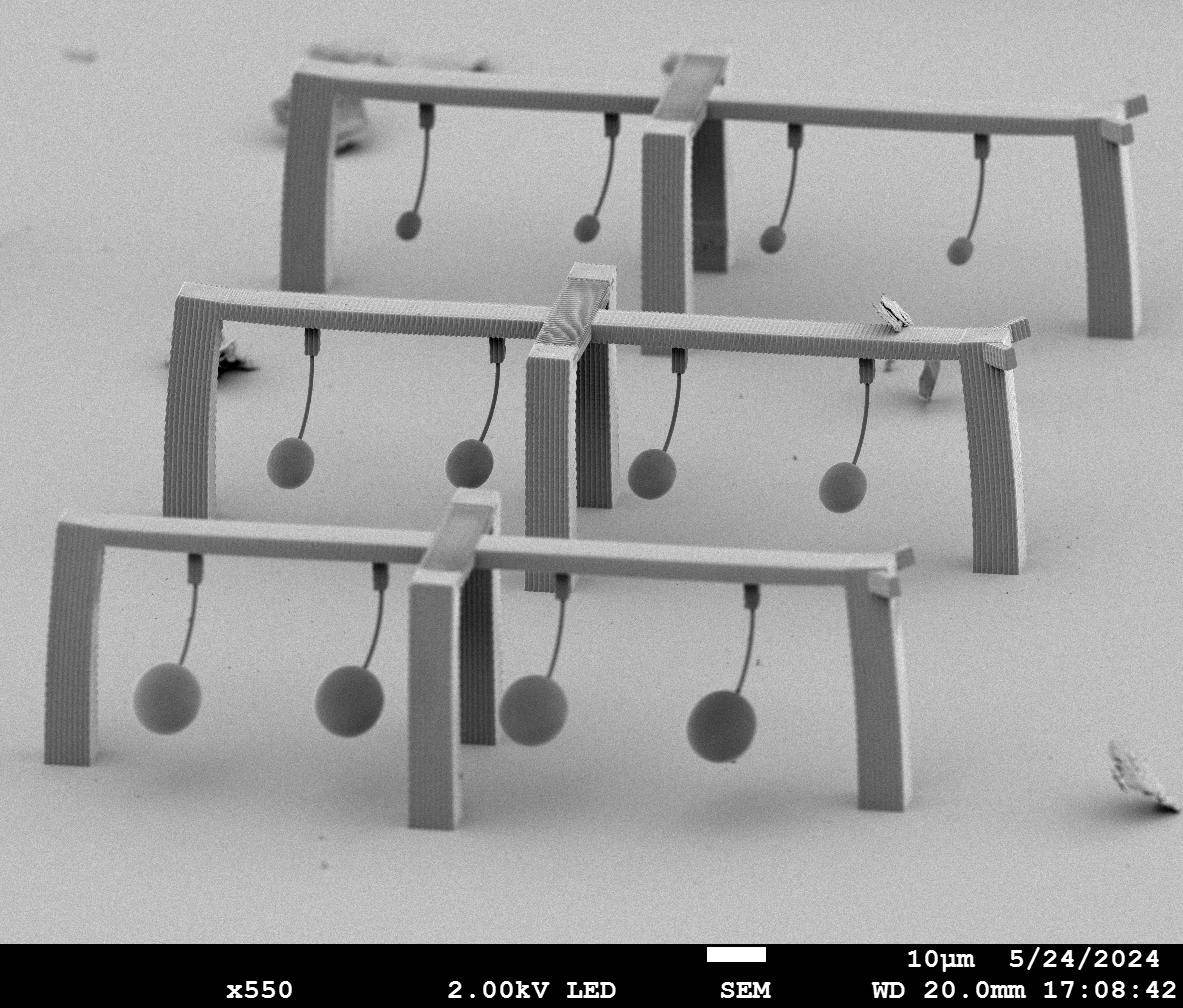

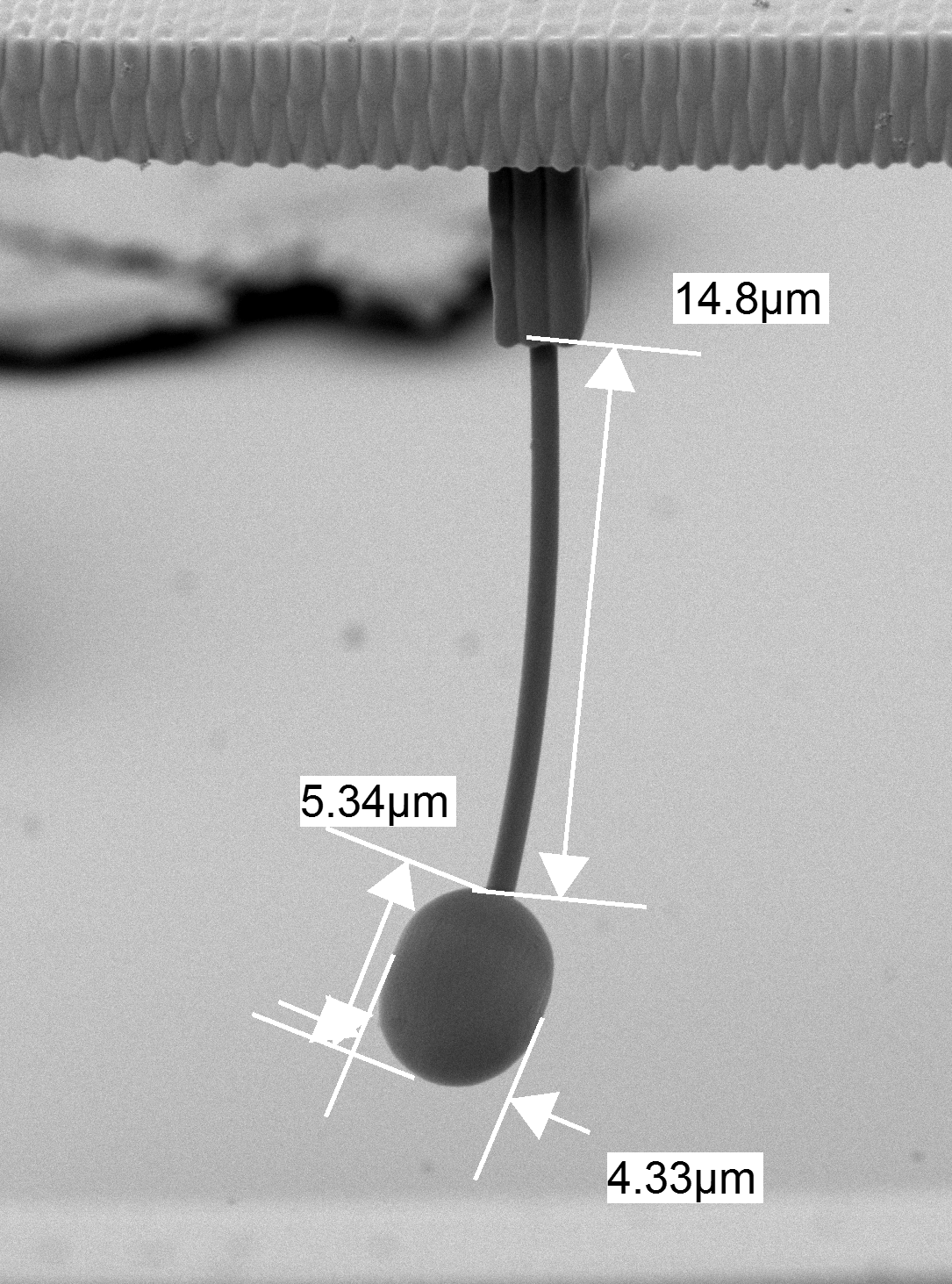


**Figure S3.** SEM images of viscometers with modified (thicker and shorter) cantilevers. The right panel shows a microbead with a designed radius of 2 µm.

**References**

[1] G. Bano *et al.*, "Power Spectral Density Analysis of Nanowire-Anchored Fluctuating Microbead Reveals a Double Lorentzian Distribution," *Mathematics,* vol. 9, no. 15, Aug 2021, Art no. 1748, doi: 10.3390/math9151748.

[2] Schaffer, E., Norrelykke, S. & Howard, J. Surface forces and drag coefficients of microspheres near a plane surface measured with optical tweezers. *Langmuir* **23**, 3654-3665 (2007).
